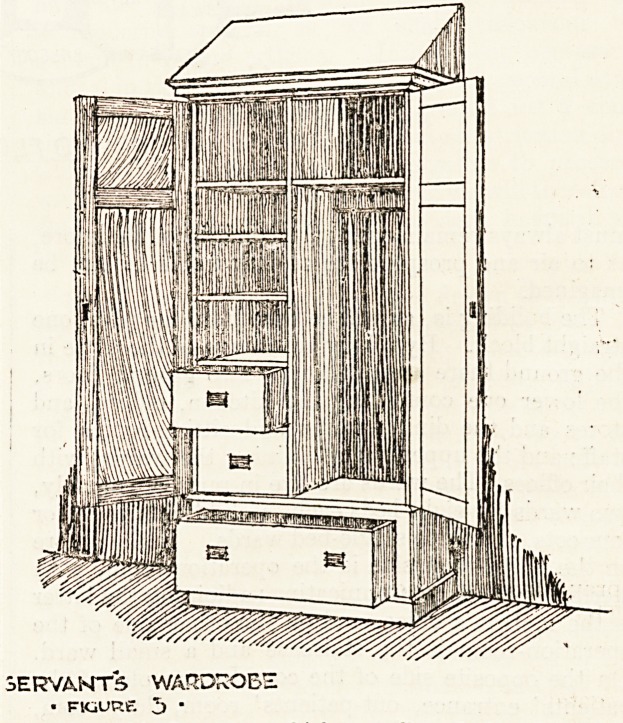# Practical Points

**Published:** 1911-10-21

**Authors:** 


### PRACTICAL POINTS.

(Criticism and Suggestions Invited.)

### Model Furniture for Staff Use.

WITH the co-operation of our readers we hope to publish an illustrated description of the best types of furniture, fittings, appliances, and so forth, at present to be met with in institutional work. We invite criticism, and shall welcome descriptive accounts with photographs or drawings of any such articles.—ED., THE HOSPITAL.

We commenced our series by publishing sketches of three types of wardrobe as used in the Manchester Royal Infirmary. Our illustration (**Fig. 1**) shows the type of wardrobe, of which twenty are provided for the use of the resident medical staff. It is of the best American ash, polished and stoutly built by a joiner, not a cabinet-maker. It contains three sliding-trays and on the right side has nine brass coat-hooks. There are two small and two large drawers, with a hat-box shelf above all. The handles are of the type known as flush-drop brass fittings. The width of the wardrobe is 4 feet 3 inches, its depth 1 foot 9 inches, and its height to underside of cornice 7 feet 4 inches.

**Fig. 2** illustrates the nurse's wardrobe, of which close on two hundred are provided. This type has a large mirror in the centre of its front. It contains two long drawers in its lower part, which are 3 feet 9 inches long

DOCTOR'S WARDROBE
• FIGURE 1 •

and 1 foot 8 inches deep; two smaller drawers are provided above 17 inches wide and of similar depth to the larger ones. In this wardrobe nine dress-hooks are provided and three sliding-trays and hat-shelves as in the doctor's wardrobe. The nurse's wardrobes are of ash, but in this case are white wax polished. The locks are all under one master-key, which controls the whole of the bedroom suite, and cup-handles are made in brass.

The servant's wardrobe is of similar design, as will be seen by reference to **Fig. 3.** In this type no sliding trays are provided, and while the two smaller drawers remain as in the other types, only one long drawer is provided, and each servant has such a wardrobe for her exclusive use. It will be noticed that the top of all these wardrobes is steeply sloped to prevent the accumulation of dust, and the Institutional staff will welcome this apt suggestion, for experience teaches that the usual ward-

NURSE'S WARDROBE
• FIGURE 2 •

robe flat top is a great dust-gatherer, which at annual cleaning time is often passed by. We are indebted to Mr. W. G. Carnt, of the Manchester Royal Infirmary, for

SERVANT'S WARDROBE
• FIGURE 3 •

details of this furniture, which was all made from his own sketches, and we hope shortly to illustrate other items of the various suites.

## Figures and Tables

**FIGURE 1 f1:**
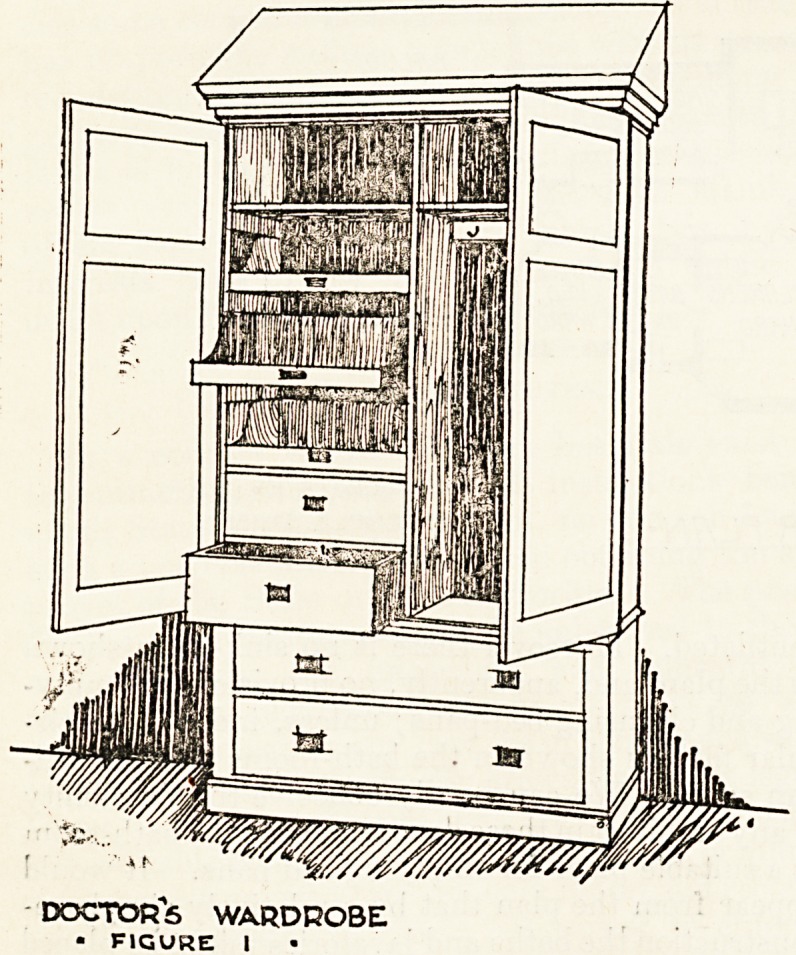


**FIGURE 2 f2:**
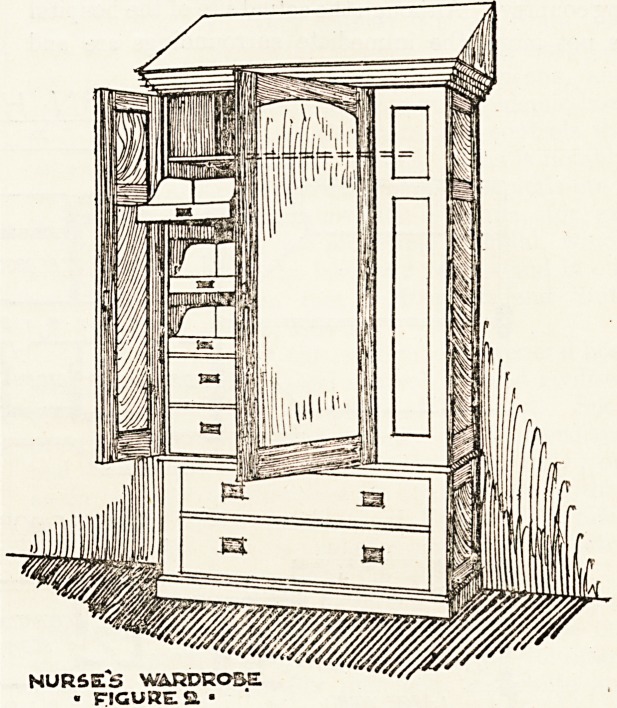


**FIGURE 3 f3:**